# Be careful of splenic rupture caused by hit by a pitch during a baseball game: a case report

**DOI:** 10.1186/s12893-021-01376-z

**Published:** 2021-10-29

**Authors:** Naoya Kobayashi, Hisao Kano, Tsukasa Kuwana, Katsuhiro Nakagawa, Masaru Matsuoka, Shingo Ihara, Nami Sawada, Junko Yamaguchi, Kosaku Kinoshita

**Affiliations:** 1 Department of Digestive Surgery, Nihon University School of Medicine, 30-1, Oyaguchikami-machi, Itabashi-ku, Tokyo, 173-8610 Japan; 2 Division of Emergency and Critical Care Medicine, Department of Acute Medicine, Nihon University School of Medicine, 30-1, Oyaguchikami-machi, Itabashi-ku, Tokyo, 173-8610 Japan

**Keywords:** Splenic rupture, Baseball, Hit by a pitch, Case report

## Abstract

**Background:**

To the best of our knowledge, splenic rupture caused by hit by a pitch (HBP) has not been previously reported. We present a patient who underwent emergency laparotomy for splenic rupture after being HBP during a baseball game.

**Case presentation:**

A 41-year-old male was HBP in the left abdomen during his first at-bat during a baseball game. During the operation, vascular injury of the splenic hilum and a deeply extending parenchymal injury were observed, and splenectomy was performed. Histologic findings were consistent with splenic rupture.

**Conclusions:**

The patient’s postoperative course was uneventful. Although extremely rare, the possibility of intra-abdominal organ injury should be considered in batters who are hit in the abdomen by a pitched baseball, as illustrated by our patient.

## Background

To the best of our knowledge, splenic rupture caused by a pitched baseball during a game has not been previously reported. We report our experience with a patient who was hit by a pitch (HBP) during a baseball game and required emergency surgery to treat a splenic rupture. Written informed consent was obtained from the patient for publication of this report and the accompanying images.

## Case report

**Fig. 1:**
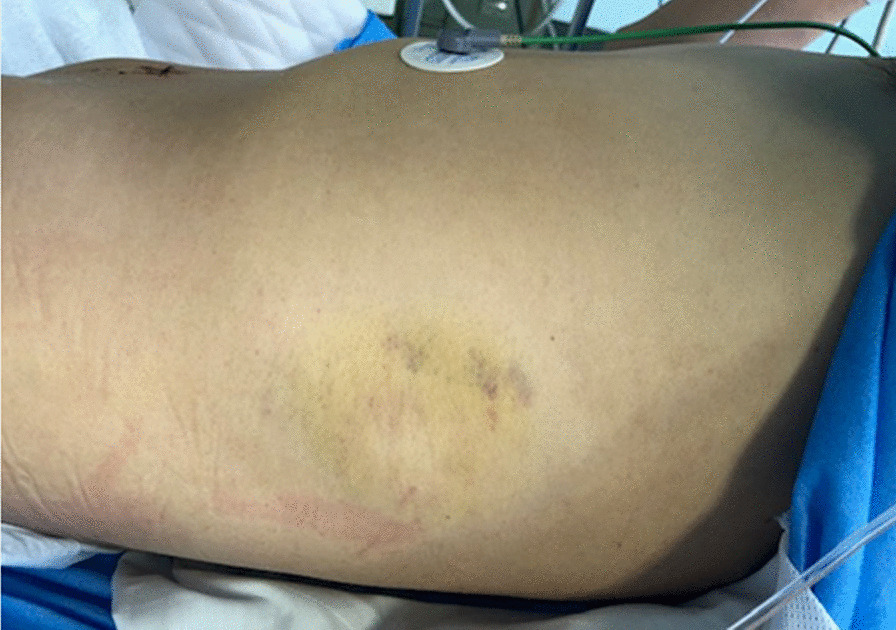
The left abdominal wall was bruised but no underlying rib fracture was present

**Fig. 2:**
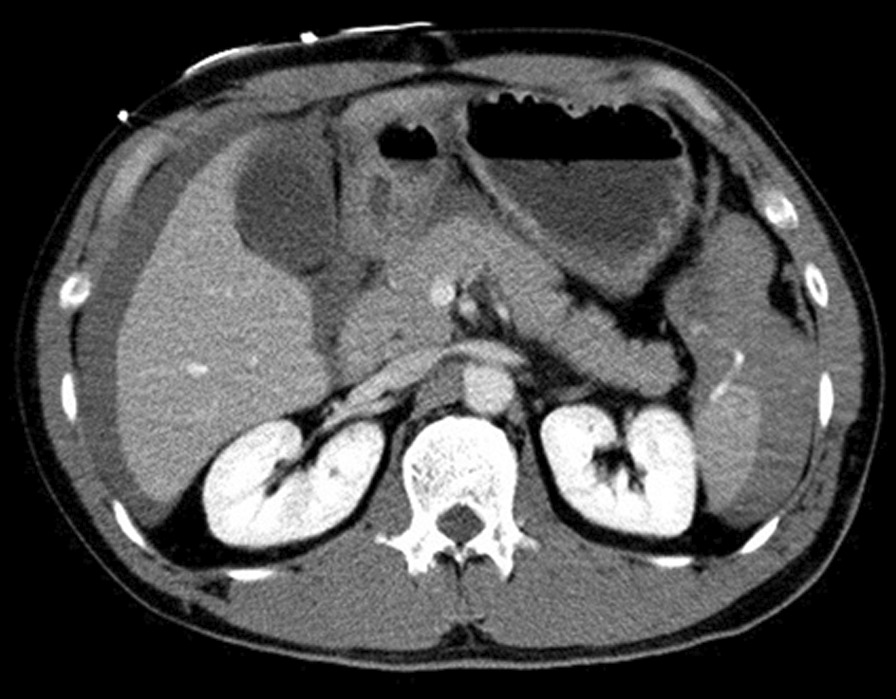
Abdominal computed tomography indicated traumatic splenic injury and intra-abdominal hemorrhage. Contrast extravasation from the lower pole of the spleen was observed

A 41-year-old male was HBP in the left abdomen during his first at-bat during a baseball game. He was standing close to the plate, and the pitcher who attempted to throw a pitch inside. The ball struck the patient and dropped directly to the ground. Although he felt pain at site of impact, the patient continued to play. However, when swinging at his second at-bat (he hit a ground ball), he experienced abdominal discomfort and subsequently removed himself from the game. After the game while traveling home on a bicycle, he lost consciousness and fell. He was transported to a local hospital where abdominal computed tomography (CT) showed a splenic injury. After transfer to our emergency and critical care center, he was pale but alert and receiving intravenous fluids. Vital signs were stable but systolic blood pressure was relatively low (80 mmHg). The injured area was bruised without underlying rib fracture (Fig. 1). CT with contrast showed traumatic splenic injury and intra-abdominal hemorrhage (Fig. 2). Emergency laparotomy was then performed. Hemorrhagic ascites was observed during the operation and approximately 2300 mL of blood was aspirated. Abdominal exploration revealed a 4-cm tear in the splenic capsule of the inferior extremity, (medial spleen) and active hemorrhage near the splenic hilum. Further exploration showed vascular injury of the hilum and a deeply extending parenchymal injury. We elected to perform splenectomy because of the severity of injury and hemorrhage. The vessels at the splenic hilum were ligated and hemostasis was achieved, prior to spleen was removal. During the operation, 1120 mL of red blood cells and 1200 mL of fresh-frozen plasma were transfused. Histopathologic examination of the spleen showed hemorrhage in the parenchyma and partial rupture of the capsule. Splenic rupture was diagnosed based on the intraoperative and histologic findings. The patient’s postoperative course was uneventful, and he was discharged home 6 days after surgery.

## Discussion

Our patient experienced no initial symptoms other than pain, at the site of baseball impact and was able to continue playing until his second at-bat. However, he later lost consciousness on his way home after the game, most likely because of hypotension due to active hemorrhage from the splenic injury. Such delayed deterioration is characteristic of sports-related intra-abdominal organ injuries [1]. Although ribs were beneath the site of impact, splenic rupture occurred without rib fracture, suggesting an extracorporeal shock wave caused the intra-abdominal injury.

Previously, traumatic splenic injuries were primarily treated surgically; however, because progress has been made in endovascular therapy, transcatheter arterial embolization (TAE) has recently contributed to saving patients’ lives in an increasing number of cases [2]. TAE is the first treatment alternative to be chosen even when hemorrhage persists, as long as blood pressure can be stabilized temporarily by fluid replacement at the time of the initial treatment. For patients who develop shock or uncontrollable hemorrhage following TAE, the treatment strategy should be switched to laparotomy immediately. Naturally, surgery should be considered for patients who do not respond to the initial infusion since the beginning of the treatment, and it is important to not miss this timing. With regard to the surgical procedure, we preserve the spleen whenever possible, considering the overwhelming post-splenectomy infections (OPSI); however, splenectomy is performed as damage control surgery without hesitation when it is considered necessary based on the extent of the splenic injury and hemodynamics. In the present case, the subject’s vital signs at the time of transportation suggested hemorrhagic shock, and CT also revealed active extravasation of the contrast medium from the inferior pole of the spleen and extensive hematoma reaching the liver surface. Because shock vitals persisted, immediate laparoscopic hemostasis was selected. As the surgical procedure, splenectomy was performed because the injury of the splenic parenchyma reached the splenic hilum and vascular injury at the splenic hilum was confirmed.

In baseball, pitched balls that hit the batter generally deflect off the body at varying angles. However, in our patient, the ball struck the left abdomen and dropped directly to the ground, indicating almost all of the ball energy was absorbed by the body. Such an event is potentially dangerous, as our patient illustrates.

Using data from the National Pediatric Trauma Registry, Wan et al. reported that abdominal trauma occurred in 459 of 5439 patients; among intra-abdominal organ injuries, injuries to the spleen were most common (50%), followed by the kidney (22%) [3]. However, to the best of our knowledge, intra-abdominal organ injury caused by a pitched baseball during a game has not been previously reported [4, 5]. In a study of 2920 HBP injuries in professional baseball players, Camp et al. found no intra-abdominal organ injuries [4].

Although equipment can be considered, to prevent injuries caused by pitched baseballs, the role of equipment remains controversial. Nicholls et al. reported that batting helmets reduce the incidence of head injuries, however, use of protective vests appears to be contraindicated [6].

## Conclusions

Although extremely rare, the possibility of intra-abdominal organ injury should be considered in batters who are hit in the abdomen by a pitched baseball. Extracorporeal shock wave-induced injuries such as splenic rupture may occur in the absence of overlying rib fracture.

## Data Availability

Not applicable.

## Abbreviations

HBP: Hit by a pitch
CT: Computed tomography
TAE: Transcatheter arterial embolization
